# Complementary and Alternative Medicine for the Treatment of Central Nervous System Disorders

**DOI:** 10.1155/2014/175152

**Published:** 2014-04-27

**Authors:** Ching-Liang Hsieh, Lixing Lao, Yi-Wen Lin, Gerhard Litscher

**Affiliations:** ^1^Acupuncture Research Center, China Medical University, Taichung 40402, Taiwan; ^2^Graduate Institute of Integrative Medicine, College of Chinese Medicine, China Medical University, Taichung 40402, Taiwan; ^3^Department of Chinese Medicine, China Medical University Hospital, Taichung 40402, Taiwan; ^4^University of Hong Kong, Hong Kong; ^5^Graduate Institute of Acupuncture Science, College of Chinese Medicine, China Medical University, Taichung 40402, Taiwan; ^6^Research Unit for Complementary and Integrative Laser Medicine, Research Unit of Biomedical Engineering in Anesthesia and Intensive Care Medicine, and TCM Research Center Graz, Medical University of Graz, 8036 Graz, Austria

Central nervous system (CNS) disorders are difficult and complicated and cause high costs for clinical therapy and basic research due to unknown and puzzling mechanisms. The treatment of CNS disorders needs systematic drugs that can pass through the brain barrier to target specific receptors. Until now, such drugs have severe side effects. Complementary and alternative medicine (CAM) has recently become highly recognized as therapeutic medicine and recommended by the World Health Organization (WHO). Clinical trials, drug development, and basic research of CAM are increased dramatically because of gradual development and knowledge. The current special issue is diversified with several novel and crucial articles concerning CAM.

Cocaine addiction is a major economic, social, and health problem in developed countries that can influence many individuals. Development of new drugs to treat cocaine dependence is urgent and necessary. Cocaine mainly binds to dopamine reuptake transporters resulting in pleasure and addiction.* Rhodiola rosea* L. (RHO) is a well-known CAM with adaptogenic, anxiolytic, antidepressive, and antistress properties. It can reduce nicotine and morphine withdrawal symptoms. F. Titomanlio et al. reported that RHO can potentiate hyperactivity induced by cocaine. RHO also attenuated the acquisition and expression of cocaine-induced conditioned place preference. They concluded that RHO is effective in decreasing the rewarding properties of cocaine but not in cocaine-associated reinstatement.

H.-C. Hsu et al. used epileptic rats to evaluate the antiepileptic effect of* Uncaria rhynchophylla* (UR) and rhynchophylline (RP). They injected kainic acid (KA) to induce seizures in Sprague Dawley rats. They suggested that pretreatment with UR and RP can reliably attenuate seizures accompanied by reduced c-Jun amino-terminal kinase phosphorylation (JNKp) of mitogen-activated protein kinase (MAPK) signal pathways in the cerebral cortex and hippocampus. IL-1*β*, IL-6, and TNF-*α* were unaltered, which means that the therapeutic effects of UR and RP are based on pJNK activation during KA-induced seizure processes. Similarly, T.-W. Guo and colleagues indicated that electroacupuncture (EA) can protect rats from chronic restraint stress. In addition, IL-1*β*, IL-6, and TGF-*β* were potentiated in chronic restraint stress rats and can be alleviated by EA pretreatment. The data are crucial that EA can attenuate depression accompanied by altering IL-1*β*, IL-6, and TGF-*β* in the hippocampal CA3 region. S.-N. Shi and colleagues reported that valtrate, which is a principle compound isolated from* Valeriana jatamansi *Jones used to treat various mood disorders, can reduce depression and simultaneously reduce the corticosterone level in the rat serum. They conclude that valtrate has an anxiolytic effect in behavioral models through the hypothalamus-pituitary-adrenal axis. The abovementioned mechanisms implied that herbal medicine and EA can activate similar mechanisms to treat CNS disorders such as depression and epilepsy.

Furthermore, K.-H. Lee et al. wanted to analyze the concurrent use of herbal medicine and hypnotic drugs in Taiwanese insomnia patients. They showed that, among 53,949 insomnia sufferers, 83.6% used hypnotic drugs. Jia-Wei-Xiao-Yao-San and Suan-Zao-Ren-Tang were always used, coadministered with hypnotic drugs. They indicated that the hazard ratio of hip fracture for hypnotic-drug users who used the herbal medicine was lower than hypnotic-drug only. The results are crucial for clinical practice to reduce hip fracture and are beneficial for health and quality of life of patients with insomnia symptoms.

CAM has wide categories to treat many diseases and symptoms. In this special issue, diverse CAM therapies are described to treat different CNS disorders such as epilepsy, depression, insomnia, and addiction. This issue is plentiful and strong in CAM therapy with evidence-based medicine from basic research to clinical results.

We think that the readers of this special issue will get many thought-provoking impulses and information.

*Ching-Liang Hsieh* *Ching-Liang Hsieh* *Lixing Lao* *Lixing Lao* *Yi-Wen Lin* *Yi-Wen Lin* *Gerhard Litscher* *Gerhard Litscher*

